# Beyond stereotypes: gendered pathways in academic self-perception and teacher identity formation in Chinese Physical Education programs

**DOI:** 10.3389/fpsyg.2025.1727557

**Published:** 2026-01-16

**Authors:** Ye Yang, Hongli Yu, Songhui You

**Affiliations:** 1 College of Physical Education and Health Science, Yibin University, Yibin, Sichuan, China; 2 School of Humanities, Tongji University, Shanghai, China; 3 College of Physical Education, Sichuan University of Science & Engineering, Zigong, Sichuan, China; 4 International College of Football, Tongji University, Shanghai, China

**Keywords:** academic self-perception, educator effectiveness, gender differences, Physical Education, professional development, self-efficacy, Social Cognitive Theory, teacher identity

## Abstract

Physical Education (PE) programs continue to face gender disparities in self-perceptions, educational effectiveness, and educator personas. Chinese educational reforms emphasize teacher quality and professional development, which necessitates a deeper understanding of how gender influences teaching identity. This study examines the relationship between academic self-perception and educator personas, emphasizing mediating aspects of educator effectiveness. We conducted a cross-sectional study with 632 undergraduate and graduate students in PE programs in Sichuan China during 2024. Participants completed validated assessments covering academic confidence, performance effort, educator effectiveness, and educator persona. Structural equation modeling was employed to analyze gender-specific pathways and evaluate both direct and indirect effects. The findings showed significant gender differences in teacher identity development. The relationship between academic confidence and educator effectiveness was stronger among men (β = 0.55**, *p* < 0.01), whereas the relationship between educator effectiveness and effort was stronger among women (β = 0.47**). Both genders showed significant associations between educator effectiveness and educator personas (β = 0.40** for women, β = 0.41** for men). Men had significantly higher variations (R^2^ = 0.39) in educator effectiveness than women (R^2^ = 0.25), suggesting gender-related identity formation pathways. Despite gender-specific routes, educator effectiveness was a key factor in educator persona formation. Findings highlight the need for gender-sensitive PE programs that address academic self-perception variations and professional development strategies. Such initiatives could enhance educator efficacy and professional identity in Chinese PE.

## 1 Introduction

Educators benefit from strong professional identities (García-Cazorla et al., 2025; Tsuda et al., 2025), which significantly influence their confidence, dedication, and classroom performance. This sense of identity is particularly significant in Physical Education (PE), where educators are expected to promote lifelong health, physical literacy, and active lifestyles (Choi et al., 2021; Yang et al., 2024). Prior research suggests that gender is associated with differences in psychological resources (Delgado-Herrera et al., 2024; Matud et al., 2020), including academic self-perception, which in turn contributes to the development of professional identity. In China, ongoing educational reforms integrate Confucian traditions with contemporary pedagogical practices, creating a context in which gender-differentiated approaches are increasingly necessary to respond to evolving social and educational expectations (Colzato et al., 2024; Wang et al., 2023). The concept of the Educator Persona (EP) reflects the dynamic interaction between personal beliefs, perceived responsibilities, and professional competencies that enable individuals to identify themselves as educators. In PE, identity development is closely linked to the acquisition of subject-specific skills, pedagogical methods, and the motivation to inspire sustained participation in physical activity (Haug et al., 2023). Higher job satisfaction, stronger resilience, and improved instructional quality are associated with well-developed teacher identities (Aydin Sünbül and Aslan Gördesli, 2021; Sun et al., 2022). However, persistent gender stereotypes that associate physical strength and leadership with masculinity and care-oriented roles with femininity continue to shape PE training experiences. Such stereotypes may create structural barriers that disproportionately affect female students entering traditionally male-dominated PE programs.

Educator Persona encompasses the behaviors, identification processes, and social categories through which individuals see themselves as educators. Professional identity is closely related to teaching practices, instructional commitment, and self-confidence in classroom settings (Nickel and Zimmer, 2019; Tsybulsky and Muchnik-Rozanov, 2019). Scholars trace EP development across educational trajectories and early professional experiences (Soomro, 2018). Empirical evidence demonstrates that EP becomes more established during teacher education and training (Casanova-Fernández et al., 2022; Simon, 2024). EP development reflects socially situated influences and individual psychological resources within pedagogical programs. Among these resources, academic self-perception and educator effectiveness (EE) are especially relevant. The Social Cognitive Theory provides an effective framework for understanding these processes in educational contexts (Schunk and DiBenedetto, 2020; Widodo and Astuti, 2024). The theory posits that individuals actively shape their development through reciprocal interactions between cognition, behavior, and the environment. Individuals who perceive themselves as capable tend to set higher goals and demonstrate greater persistence in achieving them (Teplá and Distler, 2025). In PE teacher education, EE reflects prospective teachers’ confidence in their ability to manage instruction, engage students, and meet professional demands. Such beliefs are associated with motivation, well-being, and sustained engagement during training. Recent research has documented associations between EE and EP among prospective PE educators in European contexts (Rubio-Valdivia et al., 2024), highlighting the relevance of efficacy-related beliefs for professional identity formation.

Academic self-perception refers to the set of attributes and self-evaluations individuals hold about their academic abilities and engagement. Educational research has proposed several frameworks to conceptualize self-perception in learning contexts (Chatzipanteli et al., 2025; Leo et al., 2025). At the university level, self-concept typically includes academic confidence and academic effort (Granero-Gallegos et al., 2021; Yang et al., 2023). Academic confidence reflects beliefs about capability and emotional control, while academic effort captures intrinsic motivation to invest time and energy in learning activities. Higher academic confidence has been shown to promote learning engagement and goal attainment (Xu et al., 2025; Young et al., 2025), whereas reduced confidence is associated with lower performance and increased dropout risk (Akbari and Sahibzada, 2020; Lebreton et al., 2019). Academic effort supports adjustment to university study demands and sustained academic engagement. In the Chinese context, relatively few studies have examined how gender relates to academic confidence and effort among prospective PE educators.

Although gender has received increasing attention in PE research, findings related to intrapersonal resources remain mixed. Some studies report inconclusive or inconsistent evidence regarding gender differences in self-perception and EE (Fierro-Suero et al., 2023; Frikha et al., 2024; Hermassi et al., 2023). Other PE studies have not explicitly differentiated between women and men when examining academic self-perception, EE, or EP (Granero-Gallegos et al., 2023; Nowland and Haegele, 2023). In contrast, several investigations have reported high levels of EE among men compared with women (Deng and Maria Luvimi, 2024; Preece and Bullingham, 2022; Zach et al., 2012). Additional empirical work suggests that gender may be associated with differences in teaching behaviors and professional identity development in PE (Wrench and Garrett, 2017). Research also indicated that men and women may differ in academic self-perception patterns depending on gendered learning experiences (Abós, et al., 2022). Collectively, this literature highlights the need to examine how intrinsic factors such as confidence and effort relate to professional identity formation in gender-sensitive ways (Teplá and Distler, 2025). Despite the growing recognition of gender in teacher development, substantial gaps remain in the Chinese context. First, much of the existing research on EP has been conducted in Western settings (Rubio-Valdivia et al., 2024), with limited attention to Confucian-influenced cultures that emphasize collectivism, hierarchical relationships, and social obligation. Second, while EE has been proposed as a mediator between self-perception and professional identity, little research has examined whether this mediating process differs by gender within PE training programs. Third, a gap persists between policy and practice. Although initiatives such as Healthy China 2030 emphasize PE quality improvements, teacher education curricula rarely address gender-specific challenges, which may inadvertently reproduce inequities (Schunk and DiBenedetto, 2020; Widodo and Astuti, 2024).

**FIGURE 1:**
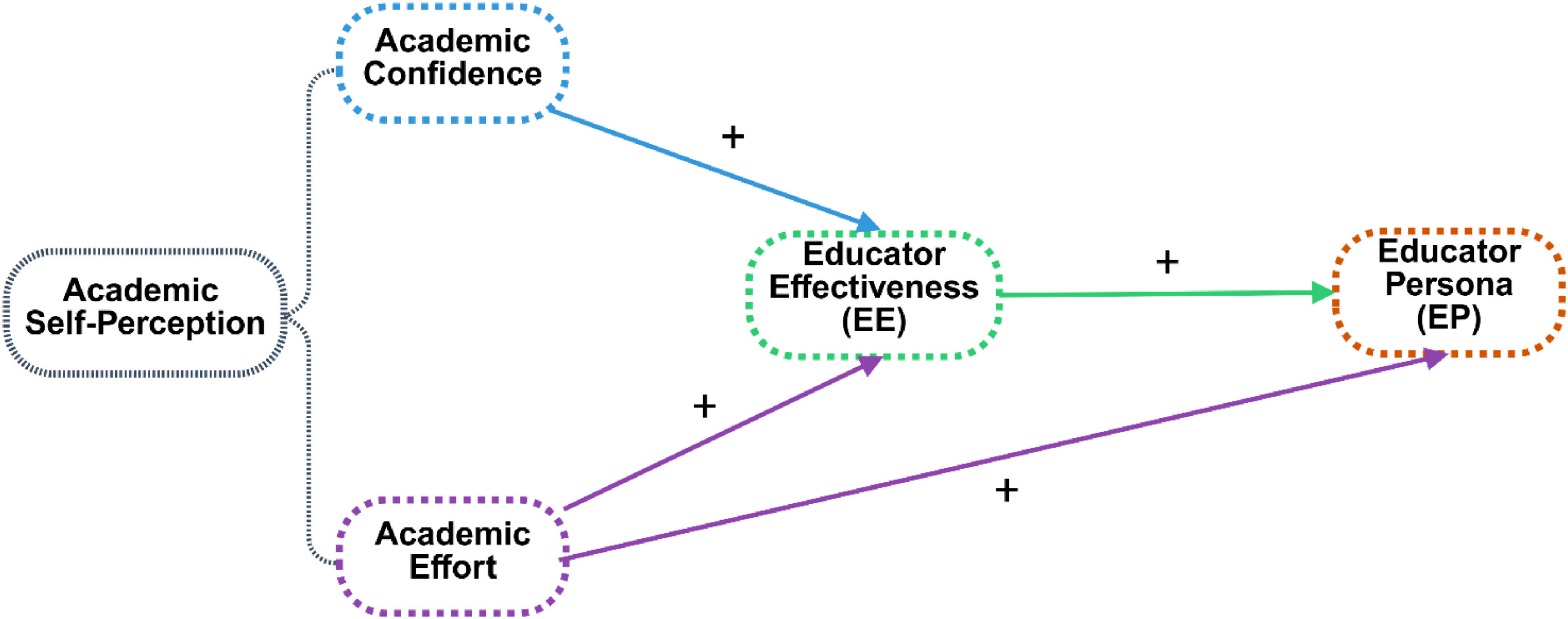
The hypothesized model for participants.

This study addresses these gaps by examining gender differences in EP among prospective PE educators in China, with particular attention to the mediating role of EE (Figure 1). The study contributes theoretically by enhancing cross-cultural understanding of professional identity development and clarifying how gender-related patterns operate within a Chinese educational context. Practically, it supports efforts to modernize teacher training by informing more equitable and responsive professional development strategies. Specifically, the study aims to (1) identify gender differences in academic self-perception, EE, and professional identity among prospective PE educators in China; (2) investigate whether EE mediates the relationship between academic self-perception and EP; and (3) examine gender differences in the associations between academic self-perception and EP. The findings are expected to inform gender-sensitive approaches to PE teacher education and professional training.

## 2 Materials and methods

### 2.1 Study area

This study was conducted in Sichuan Province, located in Southwest China (30°39′00′′N, 102°42′36′′E), a region characterized by substantial geographical, cultural, and educational diversity (Zhang et al., 2024). With a population exceeding 83 million, Sichuan is the fifth most populous province in China (National Bureau of Statistics, 2025). It serves as a major economic and educational hub in western China. The province spans the highly urbanized Chengdu Plain as well as the mountainous and ethnically diverse regions in western Sichuan. This provides a heterogeneous context for examining educational and gender-related processes. Sichuan is home to 14 officially recognized ethnic minority groups, including the Yi, Tibetan, and Qiang populations, which together account for approximately 8% of the provincial population (Chen and Suwanthada, 2024). Rapid urbanization alongside persistent rural–urban disparities has produced marked differences in educational infrastructure and resources. Although Sichuan hosts 134 higher education institutions, only 22 offer specialized PE programs (Sichuan Provincial Bureau of Statistics, 2025). Major universities, such as Chengdu Sport University and Sichuan University, provide well-established sport science and teacher education programs, whereas institutions in less-developed areas face resource constraints. Participation in PE programs in Sichuan has traditionally been male-dominated, with men comprising approximately 65%–70% of the enrolled students (National Bureau of Statistics, 2025). National and provincial policy initiatives aimed at promoting gender equity in science, technology, and vocational education have increased female participation to approximately 30%–35% over the past decade. Sichuan has also implemented localized strategies aligned with the Healthy China 2030 initiative. These strategies include the Sichuan Sports Strengthening Plan, which prioritizes improvements in the school sports infrastructure and teacher development. Despite these efforts, substantial disparities remain. Urban institutions in cities such as Chengdu and Mianyang generally possess superior facilities and access to professional development opportunities, whereas rural areas, including Liangshan and Ganzi, continue to lack basic sports amenities, which may constrain students’ academic confidence and learning engagement (Miranda and Rodriguez, 2022; Sichuan Provincial Bureau of Statistics, 2025).

Culturally, Sichuan reflects a blend of Confucian traditions and minority cultural practices, with Confucian values of hierarchy, harmony, and role differentiation continuing to shape gender expectations within educational settings (Colzato et al., 2024; Wang et al., 2023). PE contexts often encourage male trainees to demonstrate leadership and athletic competence. In contrast, female trainees are more frequently directed toward supportive or auxiliary roles. Such gender norms are particularly salient in rural and minority-dominated regions, where traditional expectations may limit women’s participation in PE training and professional advancement (Chen and Suwanthada, 2024). At the same time, urban students benefit from greater access to internships, digital learning resources, and employment opportunities, which may enhance EE and professional identity development (Long et al., 2023). These intersecting cultural, geographical, and institutional factors make Sichuan an appropriate context for examining gender-differentiated pathways linking academic self-perception, EE, and EP.

### 2.2 Research design and subjects

A cross-sectional, observational, and descriptive research design was employed in 2024. Participants were undergraduate and graduate students enrolled in initial teacher training programs with PE specialization at public universities across Sichuan Province. Eligibility criteria included: (i) active enrollment during the 2022/2023 academic year; (ii) regular attendance in face-to-face classes; (iii) completion of all questionnaire items; and (iv) provision of informed consent. Sample size estimation was conducted using G*Power software (Giner-Sorolla et al., 2024; Qiu et al., 2024). Based on a structural equation model (SEM) including four latent variables and 18 observed indicators, a minimum sample size of 500 participants was required to detect medium effect sizes (f^2^ = 0.15) with a statistical power of 0.90 at a significance level of 0.05. The final sample comprised 632 prospective PE educators, exceeding the minimum requirement for robust SEM analysis. Gender was operationalized using a binary classification (women/men), consistent with official university registration and administrative data practices in China at the time of data collection. This categorization reflects institutional and sociocultural reporting conventions rather than the assumption that gender is binary or biologically fixed. In line with contemporary educational psychology perspectives, gender is treated in this study as a contextual and socially constructed variable. It is shaped by role expectations, socialization processes, and institutional norms. The limitations of this operationalization are acknowledged in the Section “4 Discussion.”

### 2.3 Variables and metrics

Educator Persona was assessed using the unidimensional scale developed and validated by Rubio-Valdivia et al. (2024). The instrument consists of nine items (e.g., “My intention is to become an educator, which I feel confident in stating”) designed to capture individuals’ sense of vocation, professional self-actualization, and confidence in pursuing an educational career. Responses were recorded on a five-point Likert scale ranging from 1 (strongly disagree) to 5 (strongly agree). The original validation study reported excellent internal consistency [omega (ω) = 0.92]. In the present study, the scale was re-evaluated using the current sample, yielding high reliability (ω = 0.93). Confirmatory factor analysis (CFA) supported a unidimensional structure, with satisfactory model fit indices (chi-squared/degrees of freedom (χ^2^/df) = 4.51, *p* < 0.001; comparative fit index (CFI) = 0.98; Tucker–Lewis index (TLI) = 0.93; a root means square error of approximation (RMSEA) = 0.079, 90% CI [0.071, 0.091]; standardized root mean squared residual (SRMR) = 0.022).

Academic self-perception was measured using the scale developed by Granero-Gallegos et al. (2021), which includes two sub-dimensions: academic effort (three items; e.g., “While in class, my attention is focused on the teacher”) and academic confidence (three items; e.g., “It is easy for me to maintain focus during class”). Responses were collected on a seven-point Likert scale ranging from 1 (strongly disagree) to 7 (strongly agree). The original validation study reported acceptable reliability coefficients (Cronbach’s α ranging from 0.74 to 0.83). In the present study, reliability was reassessed using McDonald’s omega, yielding values of 0.81 for academic effort and 0.76 for academic confidence. CFA results indicated satisfactory model fit (χ^2^/df = 4.81, *p* < 0.001; CFI = 0.95; TLI = 0.96; RMSEA = 0.077, 90% CI [0.061, 0.112]; SRMR = 0.049), supporting the use of the scale in this context.

Educator effectiveness was assessed using the Teacher Sense of Efficacy Scale adapted for PE contexts by Burgueño et al. (2019). The instrument includes items assessing teaching strategies (four items), student engagement (four items), and classroom management (three items), rated on a nine-point Likert scale ranging from 1 (not at all) to 9 (a lot). The original validation study reported excellent reliability (α = 0.91–0.95 across subscales). In the present study, the scale was modeled as a higher-order latent construct consistent with prior research. CFA demonstrated good model fit (χ^2^/df = 4.39, *p* < 0.001; CFI = 0.96; TLI = 0.98; RMSEA = 0.076, 90% CI [0.059, 0.098]; SRMR = 0.050), and the internal consistency was excellent (ω = 0.98). No items were modified, added, or removed from any measurement instrument. References to reconceptualization pertain primarily to latent variable modeling decisions adopted to ensure theoretical coherence and comparability with previous studies.

### 2.4 Methodological workflow

Institutional approval was obtained by first contacting the department heads of undergraduate and graduate PE programs at participating universities. After institutional consent was granted, students were invited to participate via WeChat QR codes, email invitations, and follow-up phone calls. Participants received detailed information about the study objectives, procedures, anonymity protections, and their right to withdraw at any time without penalty. Questionnaires were administered online. All participants provided written informed consent prior to participation, and no minors were involved. The study was conducted in accordance with the Declaration of Helsinki and received ethical approval from Yibin University, China (approval date: December 6, 2023).

### 2.5 Statistical analysis

Internal consistency was considered satisfactory when McDonald’s omega exceeded 0.70 (Viladrich et al., 2017). CFA and SEM analyses were performed with AMOS version 29. SEM analyses followed a two-step procedure, beginning with the evaluation of the measurement model and followed by the estimation of structural relationships among the latent variables (Rubio-Valdivia et al., 2024). Model fit was assessed using χ^2^/df, CFI, TLI, RMSEA (with 90% confidence intervals), and SRMR. Values of CFI and TLI ≥ 0.90 and RMSEA and SRMR ≤ 0.08 were considered acceptable (Pan and Liu, 2024). Measurement invariance across gender groups was examined using a multi-group CFA, testing for configural, metric, and scalar invariance (Supplementary Table 1). Partial scalar invariance was established by freeing a limited number of intercepts when necessary. The data deviated from multivariate normality (Mardia coefficients: 11.79 for women and 8.39 for men, *p* < 0.001) (Pesigan and Cheung, 2020; Shi and Maydeu-Olivares, 2019); therefore, maximum likelihood estimation with bootstrapping was applied. Indirect effects were evaluated using bootstrapped confidence intervals, with significance determined when the 95% confidence interval did not include zero (Du et al., 2023; Joshi et al., 2022). Effect sizes were assessed using explained variance (R^2^), with values of approximately 0.02, 0.13, and 0.26 indicating small, medium, and large effects, respectively (Linden et al., 2024; Raykov et al., 2022). Descriptive statistics, correlations, and independent-sample *t*-tests were computed using SPSS version 29 to examine gender differences.

## 3 Results

### 3.1 The demographic characteristics of respondents

**FIGURE 2:**
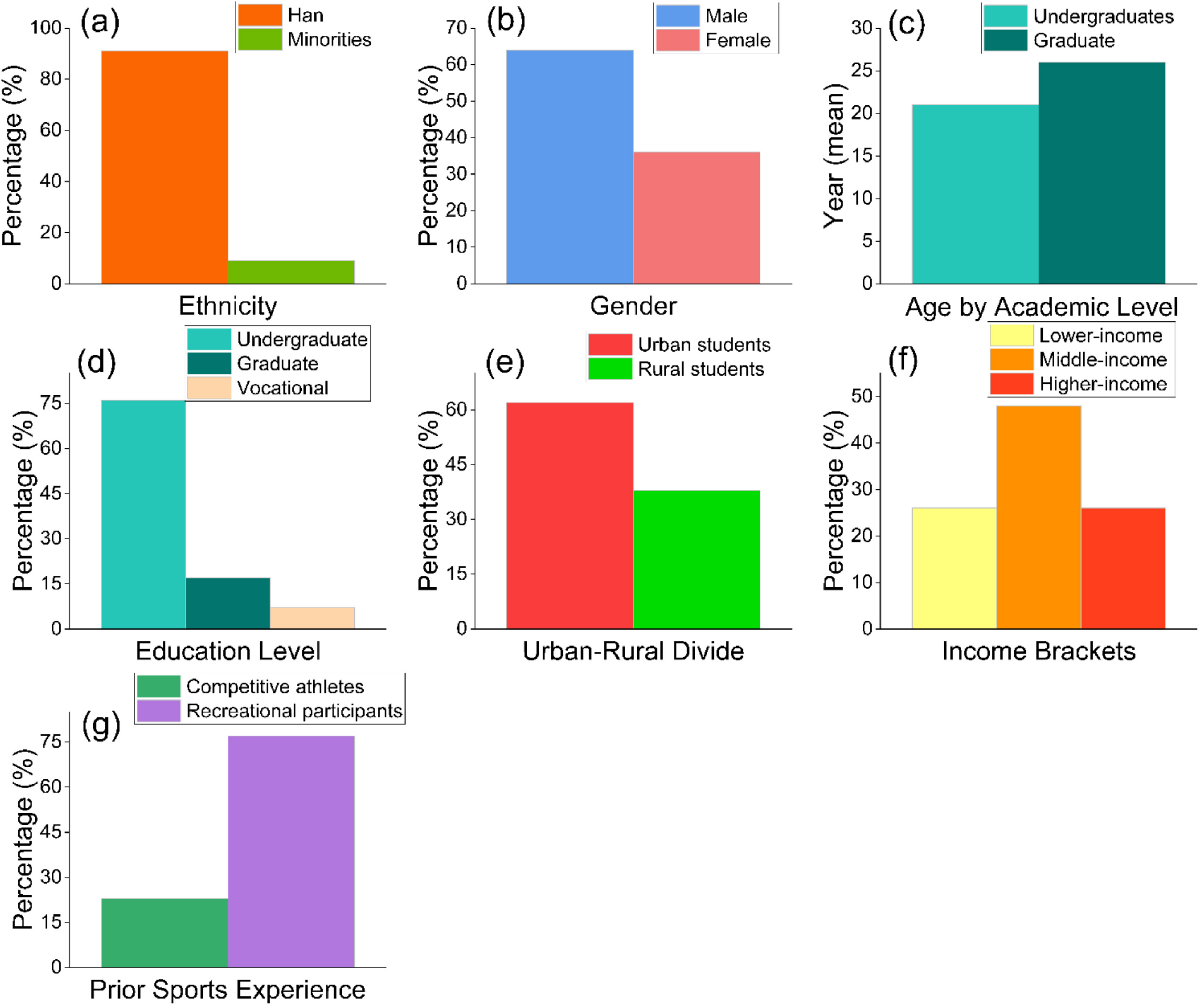
Bar graphs of demographic characteristics **(a–g)** of study participants in Sichuan, China.

The demographic characteristics of participants are summarized in Figure 2. The sample exhibited substantial diversity across ethnicity, gender, age, academic level, residential background, household income, and prior sports experience. Approximately 91% of respondents identified themselves as Han, while 9% belonged to ethnic minority groups, reflecting Sichuan Province’s demographic composition. Men comprised approximately 64% of the sample, and women accounted for 36%. This indicates a persistent gender imbalance in PE teacher education programs. Participants’ ages varied by academic level, with graduate students being older on average (approximately 26 years) than undergraduates (approximately 21 years). Undergraduate students represented the majority of the sample (76%), followed by graduate students (17%) and vocational students (7%). Participants reported urban backgrounds (62%) more than rural backgrounds (38%). Household income distribution was relatively balanced, with 48% of participants reporting middle-income status, 27% indicating higher-income status, and 25% having lower-income status. In terms of prior sports experience, approximately 77% of respondents identified themselves as recreational participants, whereas 23% reported competitive athletic experience.

These demographic patterns illustrate the heterogeneous educational and social context in which prospective PE educators in Sichuan are trained. The observed gender distribution and urban–rural differences provide important background for interpreting subsequent analyses of academic self-perception, EE, and EP, without implying causality or directional effects.

### 3.2 Preliminary findings

**FIGURE 3:**
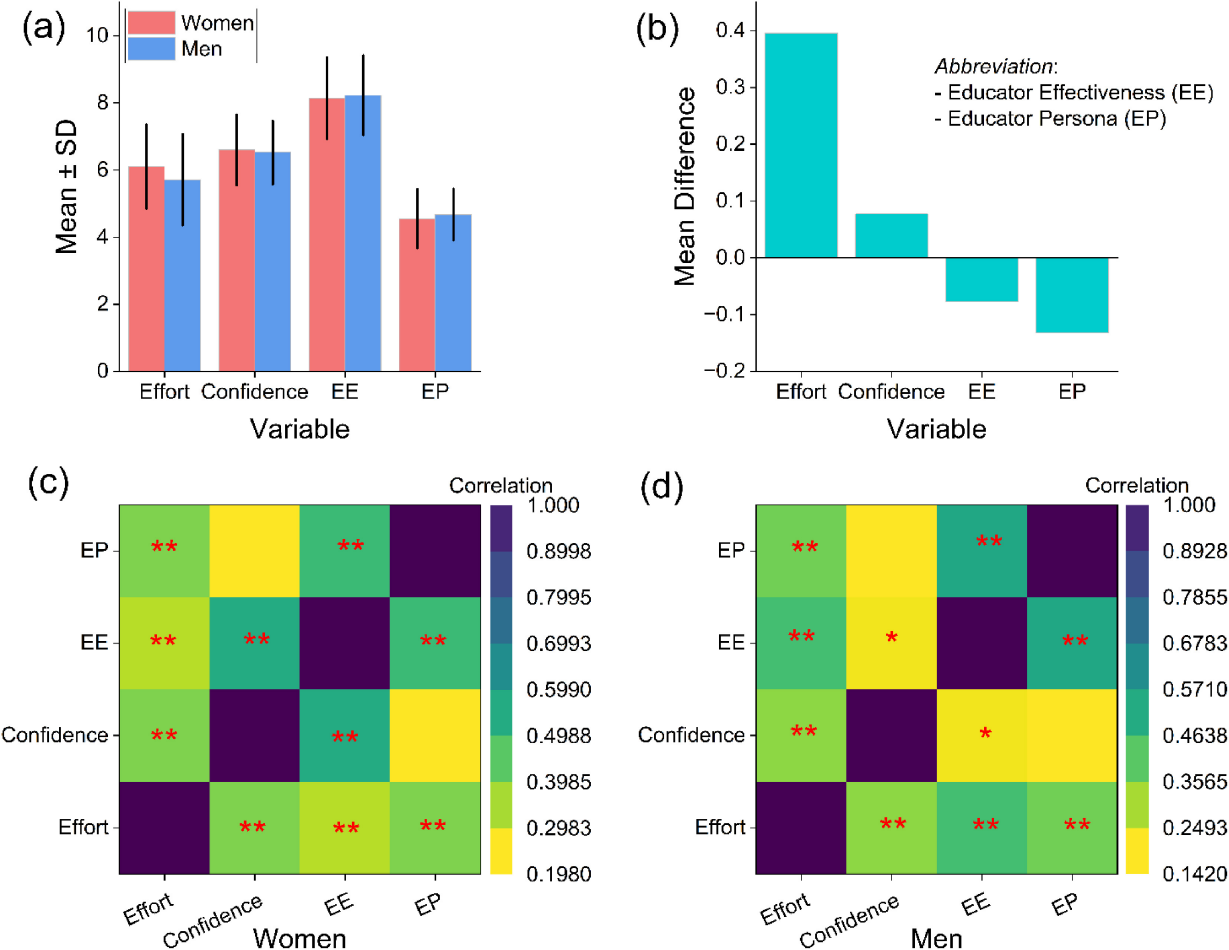
The bar graphs of descriptive statistics **(a,b)** and correlation heatmaps **(c,d)** among variables of study participants in Sichuan, China. Significant associations (*p < 0.05 and **p < 0.01).

Descriptive statistics and bivariate associations for academic effort, academic confidence, EE, and EP are presented in Figure 3. Comparisons between women and men indicated broadly similar mean levels of academic confidence (women: 6.60 ± 1.06; men: 6.52 ± 0.95) and academic effort (women: 6.10 ± 1.25; men: 5.70 ± 1.36). In contrast, men reported higher mean levels of EE (8.23 ± 1.19) and EP (4.68 ± 0.77) than women. Mean difference estimates (Figure 3b) indicated modest gender differences across variables, with larger differences observed for EE and EP than for academic self-perception dimensions.

Correlation analyses conducted separately for women and men revealed statistically significant associations among key variables (**p < 0.01), though the strength of these relationships varied by gender (Figures 3c, d). Among women, notable correlations were observed between academic effort and academic confidence (*r* = 0.286**), academic confidence and EE (*r* = 0.176**), and EE and EP (*r* = 0.484**). Among men, significant associations were identified between academic effort and academic confidence (*r* = 0.352**), academic confidence and EE (*r* = 0.506**), and EE and EP (*r* = 0.462**). Overall, both women and men exhibited interconnected patterns linking academic self-perception, EE, and EP. However, the relative strength of these associations differed, suggesting gender-differentiated configurations that warranted further examination using SEM.

### 3.3 Core findings

**FIGURE 4:**
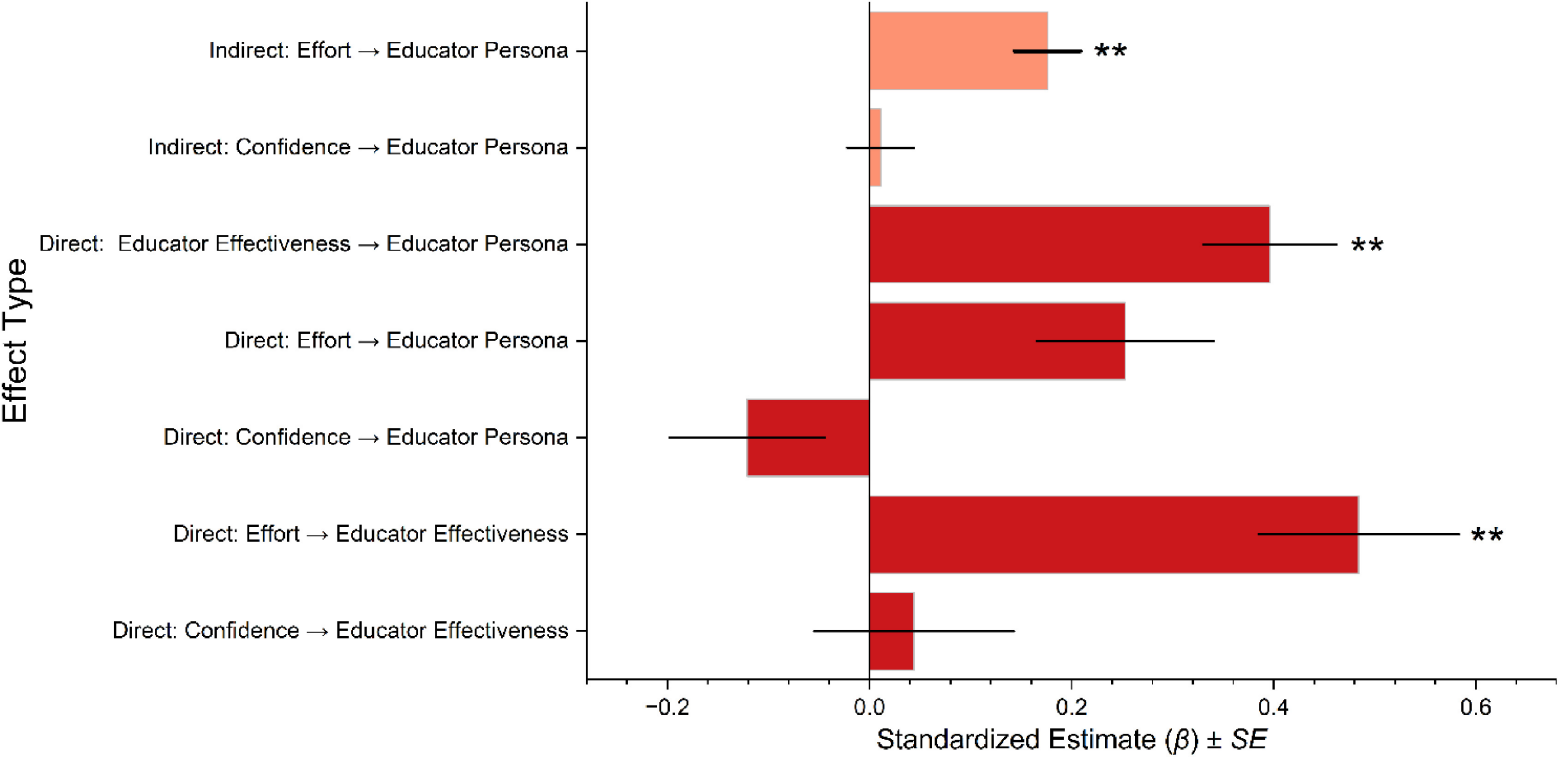
The bar graph of standardized structural equation model estimates (β) values illustrates the associations between academic confidence, academic effort, educator effectiveness, and educator persona among female participants. Significant associations (**p < 0.01).

**FIGURE 5:**
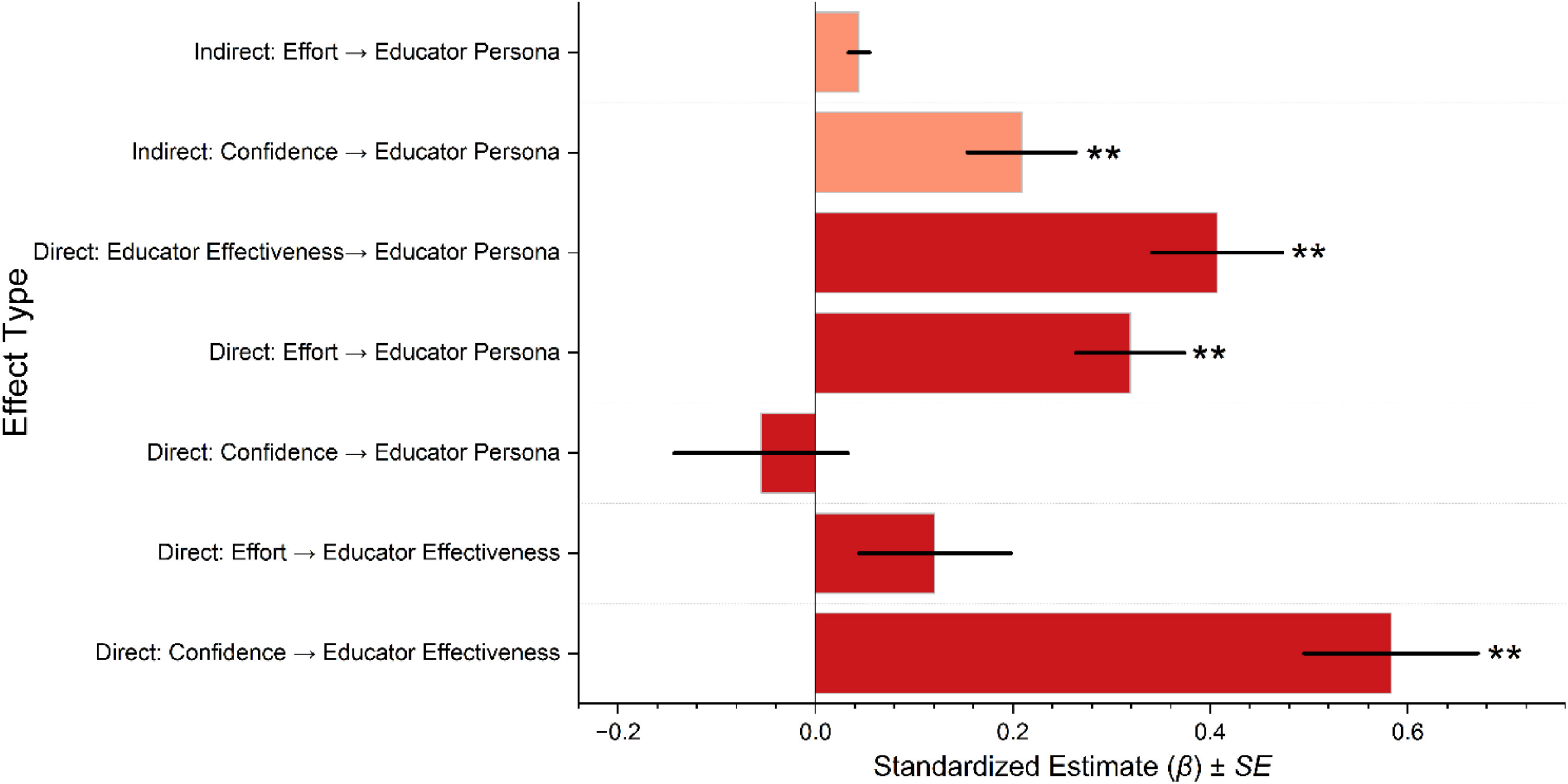
The bar graph of standardized structural equation model estimates (β) values illustrates the associations between academic confidence, academic effort, educator effectiveness, and educator persona among male participants. Significant associations (**p < 0.01).

**FIGURE 6:**
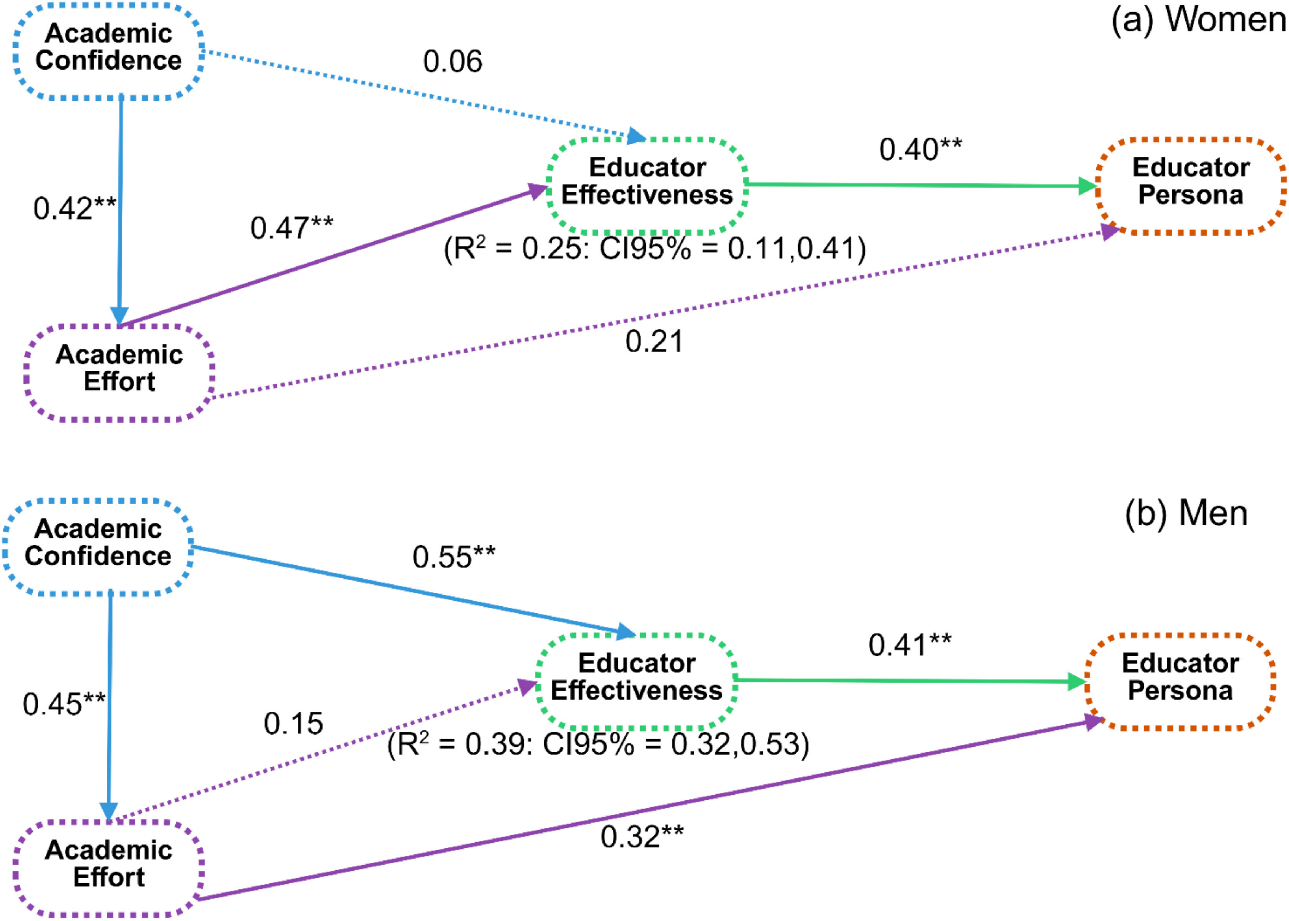
Multi-group structural equation models depict the associations between academic confidence, academic effort, educator effectiveness, and educator personas for women **(a)** and men **(b)**. Differences in pathway strengths illustrate gender-differentiated developmental patterns in teacher identity formation in Physical Education programs. Significant associations (**p < 0.01).

Structural equation model results examining associations between academic effort, academic confidence, EE, and EP are presented in Figures 4–6. All reported estimates represent standardized associations derived from cross-sectional models and therefore can be interpreted as relational rather than causal. Among women (Figure 4), EE showed the strongest association with EP (β = 0.396**, 95% CI [0.266, 0.525], **p < 0.01). Academic effort was strongly associated with EE (β = 0.484**, 95% CI [0.289, 0.678], **p < 0.01) and exhibited a small direct association with EP. Academic confidence showed a weak and non-significant direct association with EP (β = 0.044, 95% CI [−0.150, 0.238]), while its association with EE was comparatively limited. Indirect association analyses indicated that EE statistically mediated the relationship between academic effort and EP to a greater extent than between academic confidence and EP. The negative direct association observed between academic confidence and EP among women did not reach statistical significance (β = −0.121, 95% CI [−0.271, 0.029]).

Among men (Figure 5), EE was also strongly associated with EP (β = 0.407**, 95% CI [0.277, 0.536], *p* < 0.01). Academic confidence demonstrated a substantial association with EE (β = 0.583**, 95% CI [0.410, 0.755], *p* < 0.01), whereas academic effort showed a weaker and non-significant association with EE. In contrast to the pattern observed among women, academic effort displayed a statistically significant direct association with EP among men (β = 0.319**, 95% CI [0.211, 0.426], *p* < 0.01). Both academic effort and academic confidence exhibited indirect associations with EP through EE, though the magnitude of these indirect effects was smaller than the direct associations.

Figure 6 presents the multi-group structural models comparing association patterns between women and men. Among women (Figure 6a), academic confidence showed a strong association with academic effort (β = 0.42, *p* < 0.01), while its association with EE was weak and non-significant (β = 0.06). Academic effort was strongly associated with EE (β = 0.47, *p* < 0.01), and EE, in turn, was strongly associated with EP (β = 0.40, *p* < 0.01). The model explained approximately 25% of the variance in EE among women (R^2^ = 0.25, 95% CI [0.11, 0.41]). Among men (Figure 6b), academic confidence was strongly associated with both academic effort (β = 0.45, *p* < 0.01) and EE (β = 0.55, *p* < 0.01). Academic effort was directly associated with EP (β = 0.32, *p* < 0.01) but showed a weaker, non-significant association with EE. EE remained the strongest correlate of EP, with the model accounting for approximately 41% of the variance in EP and 39% of the variance in EE among men (R^2^ = 0.39, 95% CI [0.32, 0.53]). Taken together, the results indicate that EE occupies a central position in the association network linking academic self-perception and EP for both genders. However, the relative contribution of academic confidence and academic effort differs between women and men, highlighting distinct but overlapping association patterns within PE teacher education.

## 4 Discussion

This study examined gender-differentiated patterns in EP formation among prospective PE educators in China, with specific attention to the statistical role of EE in linking academic self-perception to professional identity development (Figure 3). Across both gender groups, EE emerged as the construct most strongly associated with EP. This underscores its central position in the proposed social–cognitive framework. At the same time, the strength of associations between academic confidence, academic effort, EE, and EP differed between women and men (Figure 6). Men reported higher levels of EE and EP, and their academic confidence showed a comparatively stronger association with EE. In contrast, women demonstrated a stronger association between academic effort and EE. These results indicate overlapping but distinct configurations of psychological resources related to teacher identity development within Chinese PE training contexts. The pattern that EE occupies a central role in the association network aligns with research emphasizing the importance of efficacy-related beliefs in teacher development, motivation, and professional commitment (Xu et al., 2025; Young et al., 2025). From a Social Cognitive Theory perspective, academic confidence and academic effort represent self-regulatory processes that shape how individuals evaluate competence, engage in learning activities, and persist toward professional goals (Schunk and DiBenedetto, 2020; Widodo and Astuti, 2024). In this framework, EE functions as a proximal belief structure through which academic self-perceptions become linked to a coherent EP. Importantly, these are associations derived from a cross-sectional model, and they may not be interpreted as causal or directional effects. Rather, they suggest that in PE teacher education, perceived teaching competence is closely intertwined with how prospective educators internalize professional roles (Chatzipanteli et al., 2025; Leo et al., 2025).

Gender-differentiated association patterns were evident in both descriptive and model-based results. Among men, academic confidence showed a substantial association with EE, and academic effort demonstrated a direct association with EP (Figure 6). This suggests that confidence-related competence appraisals may be particularly salient to perceived effectiveness in this group. Among women, academic effort was more strongly associated with EE, whereas academic confidence demonstrated weak direct links to EE and EP. These differences are consistent with the view that professional identity trajectories are shaped by contextually situated learning experiences, feedback processes, and role expectations within PE training environments (García-Cazorla et al., 2025; Tsuda et al., 2025). They cannot be understood as reflecting inherent or biologically fixed differences. Instead, gender-related patterns in confidence, effort, and perceived effectiveness are plausibly influenced by sociocultural norms, institutional opportunity structures, and differential reinforcement during training, especially in PE contexts where gender stereotypes remain salient (Preece and Bullingham, 2022; Wrench and Garrett, 2017). The findings also complement prior work suggesting that PE training contexts can differentially rewards or recognize competence displays and leadership behaviors, which may shape EE beliefs (Rubio-Valdivia et al., 2024). In male-dominated PE environments, men may encounter more frequent validation of competence signals associated with athletic performance or leadership-oriented roles, which can strengthen confidence–effectiveness links (Çetin and Aşkun, 2019; Horcajo et al., 2022). Conversely, women in these environments may rely more heavily on sustained effort and persistence to achieve recognition and perceived competence. This may strengthen the effort-effectiveness association. This interpretation is consistent with studies reporting that women in male-typed educational and sport-related domains often experience greater pressure to demonstrate legitimacy and work harder for equivalent recognition (Wrench and Garrett, 2017), although contextual variation is expected across institutions and local cultures.

A further implication of the multi-group results is that the explained variance in EE and EP differed across gender groups, suggesting that additional contextual variables may play a role in women’s professional identity development. For example, mentorship access, perceived belonging, stereotype threat, institutional support, and practicum quality may be particularly relevant to shaping women’s identity trajectories in PE (Abós, et al., 2022). While these factors were not directly measured in the present study, the observed association patterns indicate that expanding the model to incorporate structural and relational variables could provide a more complete explanation of gendered identity development in future works (Barrett et al., 2021; Harris et al., 2024; Safai and Krahn, 2024). Interpreting gender-related differences in educator identity formation requires ethical sensitivity. The patterns observed in this study could not be interpreted as evaluative judgments about the capabilities of women or men, nor as evidence of inherent strengths or deficits. Rather, they reflect context-dependent developmental trajectories shaped by sociocultural expectations, institutional practices, and differential socialization experiences within PE programs (Fierro-Suero et al., 2023; Frikha et al., 2024; Hermassi et al., 2023). The purpose of reporting these findings is to inform more inclusive and equitable teacher education practices, not reinforce gender stereotypes. Accordingly, gender-responsive interpretations and interventions may prioritize structural support and opportunity enhancement rather than deficit-based individual comparisons.

### 4.1 Study limitations

Several limitations may need to be considered when interpreting the findings. First, the study relied on self-reported measures, which may be influenced by social desire and response-style effects. Although validated instruments were used and their psychometric properties were re-evaluated in the current sample, future research would benefit from incorporating complementary indicators of EE, such as classroom observations, practicum evaluations, or supervisor ratings. Second, the cross-sectional design does not permit causal inferences or conclusions regarding the temporal ordering of academic self-perception, EE, and EP. The SEM pathways tested here can therefore be interpreted as theoretically informed statistical associations rather than evidence of causal mechanisms. Longitudinal designs are needed to examine whether and how confidence, effort, perceived effectiveness, and professional identity co-develop over time during PE teacher education. Third, the study was conducted in Sichuan Province, and generalizability to other regions in China or international contexts may be limited. Educational systems, cultural norms, and gender expectations in PE vary widely, and these differences may influence the structure and magnitude of the observed associations. Fourth, gender was operationalized using a binary classification consistent with administrative records, which did not capture the full spectrum of gender identities. Future studies may adopt more inclusive gender measurement approaches where institutionally and ethically feasible. Finally, the model did not include potentially relevant contextual variables (e.g., mentoring, practicum quality, institutional support, perceived belonging), which may contribute to gender-differentiated identity development. Mixed-method and qualitative approaches may be particularly useful for identifying these mechanisms and clarifying how PE training environments shape professional identity formation.

### 4.2 Implications for practice and policy

The findings have actionable implications for PE teacher education and sport-related professional development in China. First, the central role of EE suggests that teacher education programs may prioritize structured opportunities that strengthen perceived competence through progressive mastery experiences, high-quality feedback, and supportive practicum engagement. Competency-based instructional design, peer coaching, and targeted reflection practices may help trainees translate academic experiences into professional identity development. Second, the observed gender-differentiated association patterns indicate that one-size-fits-all interventions may be suboptimal. Programs may benefit from adopting gender-responsive strategies that recognize distinct developmental needs without reinforcing stereotypes. For example, where women’s EE is more strongly linked to sustained effort and persistence, programs can enhance the visibility and recognition of effort-driven achievements, provide transparent evaluation criteria, and ensure equitable access to teaching opportunities and leadership roles. Mentorship programs that connect female trainees with experienced PE educators may strengthen belonging, reduce role incongruence, and provide professional modeling that supports identity consolidation. For men, confidence-related competence beliefs appear closely connected to EE; therefore, training structures that promote reflective calibration, pedagogical skill validation, and adaptive feedback may help ensure that confidence aligns with instructional competence. Third, policy efforts aligned with Healthy China 2030 can incorporate gender-responsive professional development by embedding equity-oriented content into PE teacher training curricula, strengthening rural–urban practicum support, and providing institutional mechanisms that reduce bias in evaluation and placement. Importantly, gender-responsive interventions can be implemented with ethical reflexivity to ensure that attempts to address disparities do not inadvertently reproduce exclusionary norms. Taken together, these implications emphasize the value of designing PE teacher education environments that systematically support competence development and identity formation for all trainees, while accounting for contextual and gender-related differences in training experiences.

## 5 Conclusion

This study examined gender disparities in educator persona formation among prospective Physical Education educators in China, focusing on educator effectiveness as a mediating factor. These findings highlight distinct gender-related pathways to the development of teacher identities. Women relied more heavily on academic effort to enhance their educator effectiveness, while men possessed greater academic confidence. Regardless of gender, teaching efficacy consistently predicts professional identity, emphasizing its importance in constructing identities. The model explained more variability in educator effectiveness among men than among women, demonstrating divergent pathways for the formation of identities among men and women. Considering these results, gender-sensitive approaches are imperative in Physical Education programs to reduce academic self-perceptions and professional development disparities. Chinese Physical Education programs could benefit from specialized interventions that boost teacher effectiveness by utilizing gender-specific traits. The long-term effects of these gender-specific traits and their implications for professional development frameworks may be investigated in future research. Policymakers and educators may prioritize gender-inclusive training methodologies to ensure equitable and effective educator preparation.

## Data Availability

The raw data supporting the conclusions of this article will be made available by the authors, without undue reservation.
