# Supplementary material for: Beyond stereotypes: gendered pathways in academic self-perception and teacher identity formation in Chinese Physical Education programs
Source: Front Psychol. 2026 Jan 16;16:1727557. doi: 10.3389/fpsyg.2025.1727557 (PMC12855467; doi:10.3389/fpsyg.2025.1727557)
Supplement: Supplementary file 1 [file Table_1.docx]

Supplementary Material

# Supplementary Table

**Table S1.** Measurement invariance testing across gender (configural, metric, scalar, and partial scalar models).

| Model | χ²/df | CFI | TLI | RMSEA (90% CI) | SRMR | ΔCFI |
| --- | --- | --- | --- | --- | --- | --- |
| Configural invariance | 4.21 | 0.956 | 0.948 | 0.074 (0.068–0.081) | 0.046 | — |
| Metric invariance | 4.28 | 0.951 | 0.946 | 0.075 (0.069–0.082) | 0.049 | −0.005 |
| Scalar invariance | 4.47 | 0.937 | 0.931 | 0.079 (0.073–0.086) | 0.058 | −0.014 |
| Partial scalar invariance | 4.31 | 0.949 | 0.943 | 0.076 (0.070–0.083) | 0.051 | −0.002 |
